# Hexanal as a Predictor of Development of Oxidation Flavor in Cured and Uncured Deli Meat Products as Affected by Natural Antioxidants

**DOI:** 10.3390/foods10010152

**Published:** 2021-01-13

**Authors:** Kathrine Holmgaard Bak, Mark P. Richards

**Affiliations:** 1 Institute of Food Safety, Food Technology and Veterinary Public Health, University of Veterinary Medicine Vienna, Veterinärplatz 1, 1210 Vienna, Austria; 2 Meat Science and Animal Biologics Discovery, Department of Animal and Dairy Sciences, University of Wisconsin-Madison, 1933 Observatory Dr., Madison, WI 53706-1284, USA; mprichards@ansci.wisc.edu

**Keywords:** lipid oxidation, deli meat, hexanal, solid-phase microextraction gas chromatography, oxidation flavor, natural antioxidants, rosemary

## Abstract

Effectiveness of commercial natural antioxidants from rosemary and green tea were investigated in deli-style meat products via headspace hexanal by solid-phase microextraction gas chromatography and sensory oxidation flavor by a trained panel at weeks 1, 7, and 13 of refrigerated storage. A water/oil-soluble rosemary extract at 400 mg/kg proved the most effective antioxidant in cured deli turkey (CDT). In chicken fillet (CF), a water-soluble rosemary extract at 400 mg/kg was most efficient, especially in combination with phosphate. In pulled pork (PP), none of the antioxidants were as efficient as phosphate, though all three tested antioxidants were moderately effective in PP without phosphate. Nitrite was such an efficient antioxidant on its own in CDT that hexanal levels were so low that it was not possible to build correlation models between headspace hexanal and sensory oxidation flavor throughout the storage period. Phosphate also proved very efficient on its own in both CF and PP. It was possible to build good correlation models throughout storage for both CF and PP. Hence, hexanal was found to satisfactorily predict development of oxidation flavor in different types of uncured deli meat products both with and without added phosphate.

## 1. Introduction

Numerous natural antioxidants have been shown to be effective against lipid oxidation in a variety of meat products. In poultry, plum extract [1], green tea extract [2,3] and grape seed extract [3], sage and oregano [4], as well as pomegranate fruit juice [5] are some of the natural antioxidants found to be effective. Correspondingly, e.g., cranberry powder [6], lutein, sesamol, ellagic acid, and olive leaf extract [7], along with grape seed extract [8,9] rosemary [10,11], and green tea [10] have proven to be effective natural antioxidants in pork. Synthetic antioxidants such as butylated hydroxyanisole, butylated hydroxytoluene, and propyl gallate have been approved for use in foods by the United States Department of Agriculture, USDA, since 1955 [12]. However, safety and toxicity issues with synthetic antioxidants are leading to stricter regulations [13,14]. Consequently, consumers are increasingly demanding more natural products free from chemical preservatives [15], and, hence, employing antioxidant extracts from natural sources such as herbs, spices, seeds, fruits, and tea in meat and meat products fits well into this trend as also evidenced by a number of recent reviews and studies on the subject [16,17,18,19,20,21,22].

Phenolic compounds such as phenolic acids, phenolic diterpenes, flavonoids, and volatile oils in the natural antioxidants are responsible for the antioxidant effect [23]. Phenolic compounds can function as primary antioxidants, i.e., chain breakers, which react with free radicals generated during lipid oxidation by donation of a hydrogen atom [24]. Rosemary is among the most commonly used natural antioxidants in meat products [25] with its antioxidative effect mainly being due to phenolic diterpenes and phenolic acids [23]. Green tea owes its antioxidative effect mainly to flavonoids, especially catechin, but also tannins and vitamins [23].

Degree of lipid oxidation can be measured in a variety of ways such as peroxide value (primary lipid oxidation products) [26], thiobarbituric acid reactive substances (TBARS) (secondary lipid oxidation products) [26,27], headspace hexanal (the most abundant secondary lipid oxidation product [28]) e.g., via solid-phase microextraction gas chromatography (SPME-GC) [29], and sensory detection of oxidation [26]. Sensory methods have the advantage of detecting levels of oxidation perceivable by humans [30]. However, sensory analyses are expensive and time-consuming. Hence, being able to correlate a laboratory method with sensory analysis could save both time and money [31].

In this study, an antioxidant screening was performed by evaluating the effects of up to three different commercial natural antioxidants from rosemary (Guardian™ 09, StabilEnhance^®^ OSR D 2.5, and StabilEnhance^®^ OSR 4) and green tea (Guardian™ 20S), respectively, in three deli-style, commercially produced meat products, namely, cured deli turkey (CDT), chicken fillet (CF), and pulled pork (PP). The purpose was to determine the most efficient antioxidant and addition level for each investigated product and the potential to replace phosphate with a natural antioxidant in commercial deli-style meat products. This was done by measuring the degree of lipid oxidation in the form of headspace hexanal via SPME-GC after 1, 7, and 13 weeks of refrigerated storage and comparing the results to oxidation flavor as determined at the same time points by a trained sensory panel and determining a potential correlation between the two analyses. Performing sensory analysis is expensive and time-consuming, yet, important with regard to e.g., consumer acceptance and detection threshold [26].

## 2. Materials and Methods

### 2.1. Production of Deli Meat Products with Natural Antioxidants

All three deli meat products were thermally processed in a one-truck industrial pilot plant smokehouse using a thermal schedule designed to approximate commercial manufacturing at the company to produce a fully cooked ready-to-eat product. After processing, the products were showered to <26.7 °C and blast chilled for a short time in a −17.8 °C freezer, then stored in a cooler at 1.1–3.3 °C for 48 h prior to slicing or shredding and subsequent packaging. Addition of natural antioxidants and, for some samples, omission of phosphate (P), were the only differences to the commercially manufactured products. The natural antioxidant levels were determined based on manufacturer recommendations. The conditions of antioxidant addition and packaging for each product type were as follows:

CDT was manufactured from turkey (non-specified muscles) with one of two different types of rosemary extract (RE) at three levels each. The antioxidants were Guardian™ 09 (G09, Danisco, Madison, WI, USA) at 200, 400, and 600 mg/kg and StabilEnhance^®^ OSR D 2.5 (SE2.5, Naturex, South Hackensack, NJ, USA) at 200, 400, and 600 mg/kg as shown in Table 1. G09 is a water-soluble phenolic antioxidant containing 4% rosemary phenolic diterpenes. SE2.5 is a water- and oil-soluble deflavored plant extract from rosemary containing 2.5–3.0% carnosic acid (a phenolic diterpene). Both G09 and SE2.5 were added into the brine/water. The CDT samples were stored for up to 13 weeks refrigerated in the dark in gas flushed packaging containing 100% N_2_.

CF (chicken breast muscle) with or without sodium tripolyphosphate (0.29% Lemofos, which is a blend of 15% lemon juice solids and 85% sodium tripolyphosphate (Innophos, Inc., Cranbury, NJ, USA)) was likewise manufactured with G09 (400, 600, 800 mg/kg) and SE2.5 (400, 600, 800 mg/kg) as shown in Table 2. The Both G09 and SE2.5 were mixed with the water component prior to injection of the brine. CF samples were stored refrigerated in the dark in gas flushed packaging with an N_2_/CO_2_ ratio of 75/25. Due to mold growth, CF samples were only stored for seven weeks.

PP (pork shoulder) was manufactured with or without sodium tripolyphosphate (0.47% Lemofos) and with addition of one of three natural antioxidants, namely, Guardian™ 20S (G20S, Danisco, Madison, WI, USA) at 300, 400, and 500 mg/kg, G09 at 400, 600, and 800 mg/kg, and StabilEnhance^®^ OSR 4 (SE4, Naturex, South Hackensack, NJ, USA) as shown in Table 3. G20S is a water-soluble green tea extract containing 20% green tea catechins, which are polyphenolic flavonoids [23]. SE4 is an oil-soluble RE containing 4–5% carnosic acid. G20S was added along with the dry spices while G09 and SE4 were incorporated into the brine. The PP samples were shred after cooking on a small custom made apparatus designed to shred the meat into long fiber-containing pieces and stored for up to 13 weeks refrigerated in the dark in gas flushed packaging with an N_2_/CO_2_ ratio of 75/25.

Three batches of each sample type were prepared.

### 2.2. Solid-Phase Microextraction Gas-Chromatography (SPME-GC)

At weeks 1, 7, and 13, lipid oxidation was measured as headspace hexanal as described by [22]. Briefly, for each product, antioxidant treatment, and time-point, headspace hexanal was measured on one sample from each of the three independent batches. The SPME fiber was a 65 μm, polydimethylsiloxane–divinylbenzene (PDMS/DVB) (Supelco, Bellafonte, PA, USA), and the gas chromatograph was a HP 6890 (Hewlett-Packard, Palo Alto, CA, USA) fitted with a capillary column (DB-5, 30 m length × 0.25 mm i.d. × 0.1 μL film thickness) and a flame ionization detector. Tentative identification of hexanal was conducted by comparison to the retention time of a hexanal standard (Sigma-Aldrich, Steinheim, Germany), which was 5.43 min and the only major peak around that time point. A hexanal standard curve was prepared on a monthly basis (0.105, 0.21, 0.42, and 4.17 µg hexanal/10 µL methanol) to ensure that the system had not drifted. For details on the SPME-GC procedure, see our previous study [22].

### 2.3. Sensory Analysis

Sensory analysis was carried out by a 6-member sensory panel trained extensively according to the “Flavor Profile Method” [32]. The panel determined oxidation flavor (OF) on a scale going from 0 to 15 as described in [22]. An OF-value greater than 2–2.5 was considered necessary for sensory detection of oxidation by the sensory panel based on previous panel experience.

### 2.4. Measurement of pH

Measurement of pH was carried out in triplicate at each time point of analysis, i.e., at weeks 1, 7, and 13 (only 1 and 7 for CF) using a glass electrode (910600, Thermo Orion, Beverly, MA, USA) attached to a pH meter (Accumet AR50, Fisher Scientific, Pittsburgh, PA, USA).

### 2.5. Statistical Analysis

Statistical analysis for the effect of treatment was conducted using the software SAS^®^ (version 9.3, SAS^®^, 2002–2010). The MIXED procedure was applied to calculate least square means (LSM) and standard errors (SE) and the option Pdiff was used to calculate significant differences between LSM.

For CDT, treatment (T1 through T7) was included in the model as a fixed effect. For CF, treatment (T1 through T7), phosphate (P or nP), and the two-way interaction were included in the model as fixed effects. For PP, treatment (T1 through T10), phosphate (P or nP), and the two-way interaction were included in the model as fixed effects. *p*-values ≤ 0.05 were considered significant.

For each of the three deli meat products, the mean values for headspace hexanal were compared to OF with the purpose of creating prediction models for OF. The analysis was carried out using SAS^®^. The MIXED procedure was applied to determine if there was a significant interaction between hexanal and week (differences in slopes between weeks) or a significant effect of week (differences in intercept between weeks). *p*-values ≤ 0.05 were considered significant. In case of significance, a separate prediction model with prediction intervals was built for each week by using the proc reg statement.

## 3. Results and Discussion

### 3.1. Headspace Hexanal and pH

Hexanal content (mg/kg meat) for CDT, CF, and PP is shown in Table 1 through Table 3 with T1 in each case indicating the control sample (no antioxidant).

From looking at Table 1, it is clear that being a cured product, the level of lipid oxidation in CDT is consistently low, which is not surprising, given the antioxidant effect of nitrite, which has long been known [33]. Nevertheless, an additional positive effect of RE on lipid oxidation was found. Particularly, SE2.5 at 400 mg/kg was consistently effective at inhibiting lipid oxidation with longer storage times, though G09 at 600 mg/kg and SE2.5 at 600 mg/kg performed just as well at both week 7 and week 13.

Similarly, it is not surprising that phosphate significantly decreased lipid oxidation, which was found for both CF and PP (Table 2 and Table 3), as this effect has long been known [34]. It has previously been shown by Bak et al. [22] that a synergistic effect between phosphate and RE on lipid oxidation exists. It was hypothesized that this was due to phosphate-induced modifications in the meat structure, possibly changing the physical location of the RE within the meat product [22].

At week 1, oxidation levels in CF were lower in all samples with phosphate than samples without phosphate, regardless of antioxidant addition. At week 7, the control sample with phosphate had a hexanal level similar to those of antioxidant treated samples without phosphate. This shows that a natural antioxidant could indeed replace phosphate in chicken fillet products. Similarly, it has previously been found that rosemary at a level of 480 mg/kg could replace the synthetic antioxidant butylated hydroxyanisole (BHA) at a level of 20 mg/kg in chicken burgers during four months of frozen storage [2].

If opting to obtain the lowest possible level of oxidation in CF, the combination of phosphate and G09 seems to be the slightly better choice. Since higher antioxidant levels provided no additional benefit, G09 at 400 mg/kg would be the recommendation. G09 likewise appeared to be a slightly more efficient antioxidant than SE2.5 when no phosphate was added, and 400 mg/kg would still be the recommended level, in line with previous findings [2] (Table 2).

The difference between the two REs G09 and SE2.5 is that G09 is water-soluble and SE2.5 is water- and oil-soluble. In a low-fat product like CF (approx. 2% fat), it therefore seems reasonable that the strictly water-soluble RE, G09, would be slightly more efficient, whereas CDT contains approx. 4–5% fat and SE2.5 was the slightly more efficient antioxidant. However, this would contradict the polar paradox theory, which states that polar antioxidants are more efficient in less polar media while non-polar antioxidants are more efficient in polar media [35]. However, studies discussing the antioxidant theories beyond the polar paradox find that at low antioxidant concentrations, the effect of lipid solubility dominates with regard to antioxidant efficiency, whereas at higher concentrations, the effect of the so-called interfacial phenomenon is more important [15,35].

In PP (approx. 5–6% fat), phosphate served as a significantly more efficient antioxidant than either RE or the green tea extract G20S (Table 3). Addition of natural antioxidants had only a slight effect on the level of headspace hexanal with G20S at week 7 being the only antioxidant that was significantly effective in PP with phosphate. In PP without phosphate, there was a slight, positive effect of antioxidant addition, but with none of the antioxidants being as effective as phosphate—throughout the storage period, all antioxidant treated samples of PP without phosphate had higher levels of headspace hexanal than T1 (control) of PP with phosphate. Hence, unlike CF, it seems it may be difficult to substitute phosphate for a natural antioxidant in PP, also due to the variation in which antioxidant treatment was most efficient throughout the storage period (Table 3).

Throughout the 13-week storage period, the pH of CDT was rather stable, being 6.4 at both week 1 and week 7, and 6.3 at week 13. For CF and PP, phosphate would be expected to raise the pH of the deli meat products [36]. As it turns out, CF with phosphate had an average pH of 6.2 both at week 1 and at week 7 (no difference between control and antioxidant treated samples). CF without phosphate had an average pH of 6.1 at week 1 and 6.2 at week 7 (control 0.1 pH units lower than antioxidant treated samples at week 1). Despite the small numerical differences, the pH-values for CF with and without phosphate were shown by *t*-test to be statistically different (*p* = 0.0106). For all three storage times investigated, PP with phosphate had an average pH of 6.2, whereas PP without phosphate had an average pH of 6.0. The pH-values for PP with and without phosphate were shown by *t*-test to be statistically different (*p* < 0.0001).

Decrease in pH of meat products during storage has been related to growth of Gram positive bacteria such as lactic acid bacteria (LAB) [10,37]. The LAB content was not investigated in the present study, but the fact that only minor changes in pH were observed could indicate a good microbial quality in the form of a low amount of psychrotrophic LAB in the deli meat products in question. Psychrotrophic LAB have can cause rapid spoilage of refrigerated, cooked meat products, even of products containing nitrite [38].

### 3.2. Oxidation Flavor (OF)

CDT had an average OF of 2.5 at week 1, 2.5 at week 7, and 2.2 at week 13. There was no difference in OF between antioxidant treated samples and the control.

CF with phosphate had an average OF of 1.6 at week 1 and 1.5 at week 7, whereas CF without phosphate had an average OF of 2.0 at week 1 and 1.8 at week 7 with no practical difference between control and antioxidant treated samples. The OF-values for CF with and without phosphate were shown by *t*-test to be statistically different (*p* < 0.0001). G09 was the most efficient antioxidant in CF concerning reduction in headspace hexanal with 400 mg/kg being the recommended level of addition. Numerically, SE2.5 had 0–0.3-unit lower OF-values than G09 for the same combination of phosphate and storage time, though this difference is so small that it likely has no practical significance.

PP with phosphate had an average OF of 1.8 at week 1, 2.2 at week 7, and 2.1 at week 13, whereas PP without phosphate had an average OF of 2.3 at week 1, 2.7 at week 7, and 3.1 at week 13. The OF-values for PP with and without phosphate were shown by *t*-test to be statistically different (*p* < 0.0001).

It should be noted that none of the above-mentioned OF-values were enough to lead to rejection by the sensory panel as the values were even below or just above the level of OF detection of 2–2.5. Similar results were found by [22] when using hexanal as a marker for oxidation in sliced, uncured deli turkey with added RE.

Looking at the correlation between headspace hexanal determined by SPME-GC and OF determined by the sensory panel, it turns out that, for CDT, there was a significant effect of week (*p* = 0.005), while for CF and PP, there were significant interactions between headspace hexanal and week (*p* = 0.038 for CF; *p* = 0.016 for PP). Hence, for all three product types, correlation models were built separately for each week. Significance of correlation between headspace hexanal and oxidation flavor for CDT, CF, and PP after 1, 7, and 13 weeks of refrigerated storage is given in Table 4.

The correlation model for CDT was only good at week 7. The very low headspace hexanal level is likely the cause for this lack of correlation, as the sensory *OF*-values for CDT were on par with those for PP.

It was possible to build, statistically speaking, good correlation models for both CF and PP for all the weeks of refrigerated storage studied.

**Figure 1:**
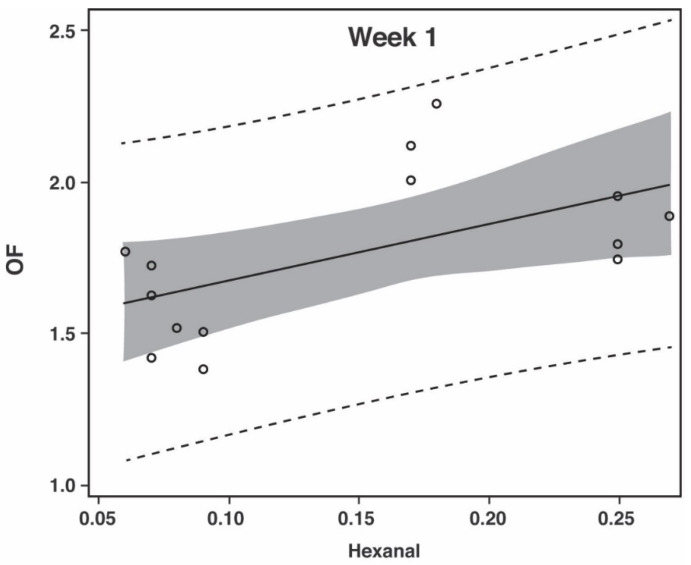

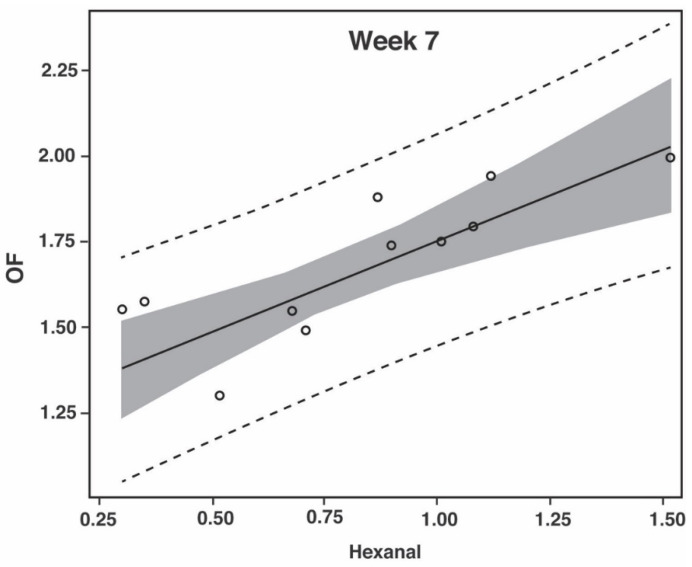
Prediction plot for off-flavor (OF) from headspace hexanal when examining chicken fillet at week 1 (*R*^2^ = 0.332; *p* = 0.031) and week 7 (*R*^2^ = 0.649; *p* = 0.001) of refrigerated storage. Dashed lines provide the 95% prediction limits. The shaded area is the 95% confidence limits of the fit. The finished product was thermally processed and modified atmosphere packaged in 75% N_2_ and 25% CO_2_. The off-flavor scale was 0 to 15 with 15 being highest off-flavor. Units for hexanal were mg per kg (weight/weight).

The prediction models for CF illustrated in Figure 1 have the following Equations (1) and (2), with the 95% prediction intervals (PI) in parentheses:

(1) $$\mathrm{Week}\;1:\;OF=1.86\times hexanal+1.49\;\left(\pm 0.54\right),\;p=0.031,\;R^{2}=0.332$$

(2) $$\mathrm{Week}\;7:\;OF=0.54\times hexanal+1.21\;\left(\pm 0.36\right),\;p=0.001,\;R^{2}=0.649$$

**Figure 2:**
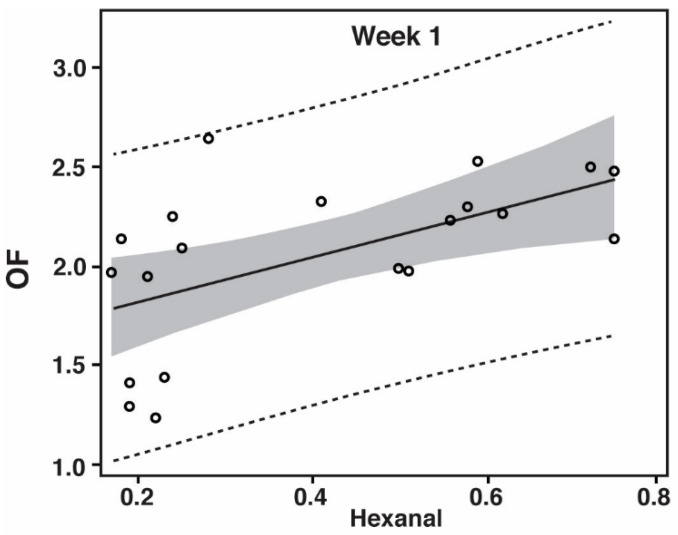

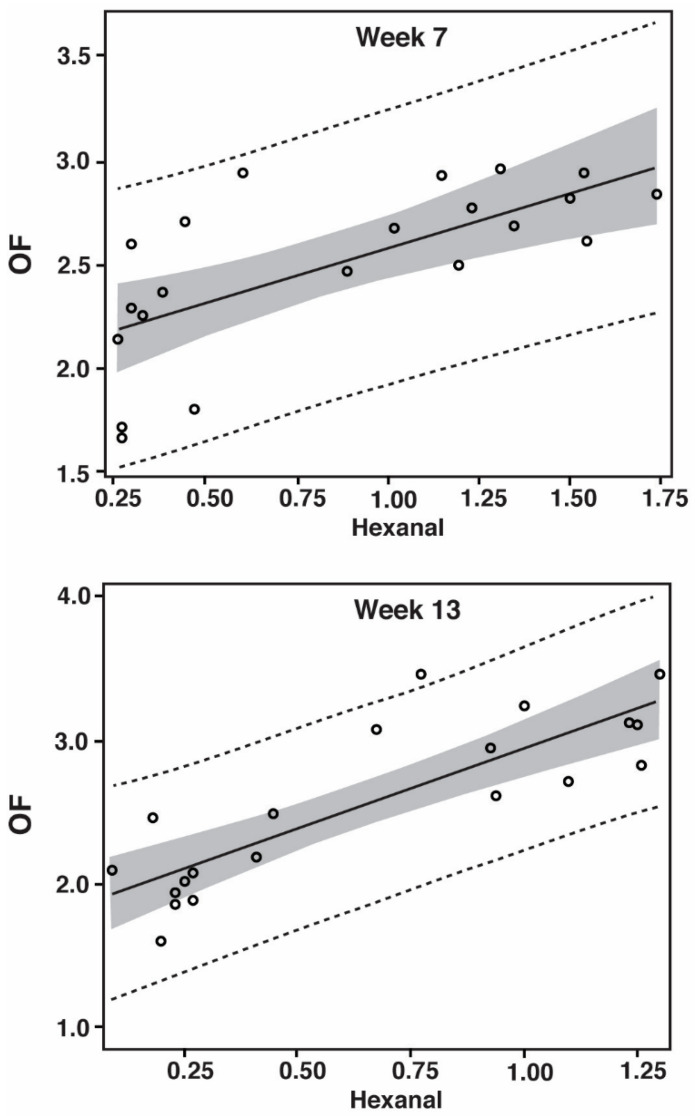
Prediction plot for off-flavor (OF) from headspace hexanal when examining pulled pork at week 1 (*R*^2^ = 0.338; *p* = 0.007), week 7 (*R*^2^ = 0.468; *p* = 0.001), and week 13 (*R*^2^ = 0.701; *p* < 0.0001) of refrigerated storage. Dashed lines provide the 95% prediction limits. The shaded area is the 95% confidence limits of the fit. The finished product was thermally processed and modified atmosphere packaged in 75% N_2_ and 25% CO_2_. The off-flavor scale was 0 to 15 with 15 being highest off-flavor. Units for hexanal were mg per kg (weight/weight).

The prediction models for PP illustrated in Figure 2 have the following Equations (3)–(5), with the 95% prediction intervals (PI) in parentheses:

(3) $$\mathrm{Week}\;1:\;OF=1.13\times hexanal+1.60\;\left(\pm 0.79\right),\;p=0.007,\;R^{2}=0.338$$

(4) $$\mathrm{Week}\;7:\;OF=0.52\times hexanal+2.05\;\left(\pm 0.70\right),\;p=0.001,\;R^{2}=0.468$$

(5) $$\mathrm{Week}\;13:\;OF=1.11\times hexanal+1.84\;\left(\pm 0.73\right),\;p<0.0001,\;R^{2}=0.701$$

However, by looking at Figure 1 and Figure 2 it is evident that the correlation models are best suited for the later stages of storage (after week 1), which can be explained by the fact that hexanal, being a secondary lipid oxidation product [28], is not present in large quantities at the early stages of oxidation. This is especially true for the phosphate containing samples where the oxidation levels are being kept especially low (Table 2 and Table 3). Nevertheless, when using hexanal as an indicator of lipid oxidation and meat flavor deterioration, one must be aware of the further reaction of hexanal [39], which may make it a less optimal indicator compound at later stages of oxidation for some food products.

The results of these correlation studies are in line with previously reported results for sliced, uncured deli turkey with or without phosphate and with added RE [22], cooked ground pork [40], cooked beef [41], and a meat model system made from boneless pork loins [39].

## 4. Conclusions

The effectiveness of up to three different commercial natural antioxidants from rosemary and green tea, respectively, were investigated in three deli-style meat products by determining headspace hexanal by SPME-GC and sensory oxidation flavor by a trained panel at weeks 1, 7, and 13 of refrigerated storage. The water- and oil-soluble RE SE2.5 at 400 mg/kg proved the most effective antioxidant in cured deli turkey. In chicken fillet, the water-soluble RE G09 at 400 mg/kg was the most efficient antioxidant, especially in combination with phosphate. In pulled pork, none of the antioxidants were as efficient as phosphate, though the water-soluble green tea extract G20S resulted in decreased headspace hexanal level at week 7 in PP with phosphate, while G20S, R09, and the oil-soluble RE SE4 were all moderately effective antioxidants in PP without phosphate. Nitrite was such an efficient antioxidant on its own in CDT that hexanal levels were so low that it was not possible to build a correlation model between headspace hexanal and sensory oxidation flavor throughout the storage period. Phosphate proved very efficient on its own, but with an additional effect of the natural antioxidants. It was possible to build good correlation models between headspace hexanal and oxidation flavor throughout storage for both CF and PP, most efficiently after week 1 of storage. Hence, hexanal was found to satisfactorily predict development of oxidation flavor in different types of uncured deli meat products both with and without added phosphate.

## Figures and Tables

**Table 1:** Hexanal content (mg/kg meat) in cured deli turkey. Estimate indicates the mean hexanal content for triplicate determinations. Means with different letters within each week indicate significant difference (*p* ≤ 0.05) between treatments.

Treatment	Antioxidant	Level (mg/kg)	Hexanal (mg/kg Meat)					
			Week 1		Week 7		Week 13	
			Estimate	SE	Estimate	SE	Estimate	SE
T1	Control	0	0.14 a	0.01	0.16 a	0.02	0.16 a	0.01
T2	Guardian 09	200	0.09 b	0.01	0.13 ab	0.02	0.13 ab	0.01
T3	Guardian 09	400	0.08 b	0.01	0.14 ab	0.02	0.10 b	0.01
T4	Guardian 09	600	0.09 b	0.01	0.11 bc	0.02	0.09 bc	0.01
T5	StabilEnhance OSR D 2.5	200	0.08 b	0.01	0.11 bc	0.02	0.10 b	0.01
T6	StabilEnhance OSR D 2.5	400	0.08 b	0.01	0.08 c	0.02	0.06 c	0.01
T7	StabilEnhance OSR D 2.5	600	0.08 b	0.01	0.10 bc	0.02	0.10 bc	0.01

**Table 2:** Hexanal content (mg/kg meat) in chicken fillet with or without phosphate. Estimate indicates the mean hexanal content for triplicate determinations. Means with different letters within each week indicate significant difference (*p* ≤ 0.05) between treatments. P = samples containing phosphate, nP = samples without phosphate.

Treatment		Antioxidant	Level (mg/kg)	Hexanal (mg/kg Meat)			
				Week 1		Week 7	
				Estimate	SE	Estimate	SE
**P**	T1	Control	0	0.07 c	0.01	0.81 bcde	0.17
	T2	Guardian 09	400	0.07 c	0.01	0.23 g	0.17
	T3	Guardian 09	600	0.06 c	0.01	0.35 efg	0.17
	T4	Guardian 09	800	0.07 c	0.01	0.30 fg	0.17
	T5	StabilEnhance OSR D 2.5	400	0.09 c	0.01	0.71 cdef	0.17
	T6	StabilEnhance OSR D 2.5	600	0.09 c	0.01	0.68 cdefg	0.17
	T7	StabilEnhance OSR D 2.5	800	0.08 c	0.01	0.52 defg	0.17
**nP**	T1	Control	0	0.27 a	0.01	1.52 a	0.17
	T2	Guardian 09	400	0.17 b	0.01	0.87 bcd	0.17
	T3	Guardian 09	600	0.17 b	0.01	0.90 bcd	0.17
	T4	Guardian 09	800	0.18 b	0.01	1.01 bc	0.17
	T5	StabilEnhance OSR D 2.5	400	0.25 a	0.01	1.12 abc	0.17
	T6	StabilEnhance OSR D 2.5	600	0.25 a	0.01	1.08 abc	0.17
	T7	StabilEnhance OSR D 2.5	800	0.25 a	0.01	1.25 ab	0.17

**Table 3:** Hexanal content (mg/kg meat) (mean of three samples) in pulled pork with or without phosphate. Estimate indicates the mean hexanal content for triplicate determinations. Means with different letters within each week indicate significant difference (*p* ≤ 0.05) between treatments. P = samples with phosphate, nP = samples without phosphate.

Treatment		Antioxidant	Level (mg/kg)	Hexanal (mg/kg meat)					
				Week 1		Week 7		Week 13	
				Estimate	SE	Estimate	SE	Estimate	SE
**P**	T1	Control	0	0.19 fg	0.04	0.47 hi	0.06	0.27 gh	0.06
	T2	Guardian 20S	300	0.22 fg	0.04	0.27 jk	0.06	0.20 h	0.06
	T3	Guardian 20S	400	0.19 fg	0.04	0.27 jk	0.06	0.23 h	0.06
	T4	Guardian 20S	500	0.23 fg	0.04	0.26 k	0.06	0.23 h	0.06
	T5	Guardian 09	400	0.24 fg	0.04	0.44 hij	0.06	0.09 h	0.06
	T6	Guardian 09	600	0.18 g	0.04	0.30 ijk	0.06	0.18 h	0.06
	T7	Guardian 09	800	0.28 f	0.04	0.60 h	0.06	0.46 f	0.06
	T8	StabilEnhance OSR 4	400	0.25 fg	0.04	0.33 ijk	0.06	0.41 fg	0.06
	T9	StabilEnhance OSR 4	600	0.21 fg	0.04	0.38 ijk	0.06	0.27 gh	0.06
	T10	StabilEnhance OSR 4	800	0.17 g	0.04	0.30 ijk	0.06	0.25 gh	0.06
**nP**	T1	Control	0	0.75 a	0.04	1.55 b	0.06	1.26 ab	0.06
	T2	Guardian 20S	300	0.51 d	0.04	1.20 de	0.06	0.94 cd	0.06
	T3	Guardian 20S	400	0.50 de	0.04	1.02 fg	0.06	1.10 bc	0.06
	T4	Guardian 20S	500	0.41 e	0.04	0.89 g	0.06	1.23 ab	0.06
	T5	Guardian 09	400	0.58 cd	0.04	1.15 ef	0.06	1.00 c	0.06
	T6	Guardian 09	600	0.59 cd	0.04	1.50 bc	0.06	1.25 ab	0.06
	T7	Guardian 09	800	0.56 cd	0.04	1.31 de	0.06	0.77 de	0.06
	T8	StabilEnhance OSR 4	400	0.62 bc	0.04	1.54 b	0.06	0.67 e	0.06
	T9	StabilEnhance OSR 4	600	0.75 a	0.04	1.35 cd	0.06	1.30 a	0.06
	T10	StabilEnhance OSR 4	800	0.72 ab	0.04	1.74 a	0.06	0.93 cd	0.06

**Table 4:** Significance of correlation between headspace hexanal and oxidation flavor for cured deli turkey (CDT), chicken fillet (CF), and pulled pork (PP) after 1, 7, and 13 weeks of refrigerated storage. —indicates that no measurements were done for CF at week 13.

Significance of Correlation between Hexanal and Oxidation Flavor			
	Week 1	Week 7	Week 13
CDT	*p* = 0.7960	*p* = 0.0379	*p* = 0.4269
CF	*p* = 0.0312	*p* = 0.0005	---
PP	*p* = 0.0071	*p* = 0.0009	*p* < 0.0001

## Data Availability

The data presented in this study are available on request from the corresponding author.

## Abbreviations

BHA = butylated hydroxyanisole; CDT = cured deli turkey; CF = chicken fillet; G09 = Guardian™ 09; G20S = Guardian™ 20S; LAB = lactic acid bacteria; LSM = least square means; nP = samples without phosphate; OF = oxidation flavor; P = samples with phosphate; PP = pulled pork; RE = rosemary extract; SE = standard error; SE2.5 = StabilEnhance^®^ OSR D 2.5; SE4 = StabilEnhance^®^ OSR 4; SPME-GC = solid-phase microextraction gas chromatography; T = treatment; TBARS = thiobarbituric acid reactive substances; USDA = United States Department of Agriculture
